# Summary of clinical investigation plan for The DIATEC trial: in-hospital diabetes management by a diabetes team and continuous glucose monitoring or point of care glucose testing – a randomised controlled trial

**DOI:** 10.1186/s12902-024-01595-4

**Published:** 2024-05-06

**Authors:** Mikkel Thor Olsen, Carina Kirstine Klarskov, Ulrik Pedersen-Bjergaard, Katrine Bagge Hansen, Peter Lommer Kristensen

**Affiliations:** 1 Department of Endocrinology and Nephrology, Copenhagen University Hospital – North Zealand, Hilleroed, Denmark; 2 Steno Diabetes Center Copenhagen, Copenhagen University Hospital – Herlev-Gentofte, Herlev, Denmark; 3 Department of Clinical Medicine, Faculty of Health and Medical Sciences, University of Copenhagen, Copenhagen, Denmark

**Keywords:** In-hospital, Inpatient, Diabetes, Continuous glucose monitoring, RCT

## Abstract

**Background:**

Worldwide, up to 20 % of hospitalised patients have diabetes mellitus. In-hospital dysglycaemia increases patient mortality, morbidity, and length of hospital stay. Improved in-hospital diabetes management strategies are needed. The DIATEC trial investigates the effects of an in-hospital diabetes team and operational insulin titration algorithms based on either continuous glucose monitoring (CGM) data or standard point-of-care (POC) glucose testing.

**Methods:**

This is a two-armed, two-site, prospective randomised open-label blinded endpoint (PROBE) trial. We recruit non-critically ill hospitalised general medical and orthopaedic patients with type 2 diabetes treated with basal, prandial, and correctional insulin (*N* = 166).

In both arms, patients are monitored by POC glucose testing and diabetes management is done by ward nurses guided by in-hospital diabetes teams. In one of the arms, patients are monitored in addition to POC glucose testing by telemetric CGM viewed by the in-hospital diabetes teams only. The in-hospital diabetes teams have operational algorithms to titrate insulin in both arms. Outcomes are in-hospital glycaemic and clinical outcomes.

**Discussion:**

The DIATEC trial will show the glycaemic and clinical effects of in-hospital CGM handled by in-hospital diabetes teams with access to operational insulin titration algorithms in non-critically ill patients with type 2 diabetes. The DIATEC trial seeks to identify which hospitalised patients will benefit from CGM and in-hospital diabetes teams compared to POC glucose testing. This is essential information to optimise the use of healthcare resources before broadly implementing in-hospital CGM and diabetes teams.

**Trial registration:**

Prospectively registered at ClinicalTrials.gov with identification number NCT05803473 on March 27^th^ 2023.

**Supplementary Information:**

The online version contains supplementary material available at 10.1186/s12902-024-01595-4.

## Introduction

Worldwide, up to 20% of hospitalised patients have diabetes mellitus [1–3]. In hospital diabetes management is insufficiently researched [4] and can be inadequate [3, 5–9]. Dysregulated glucose levels might increase patient mortality, morbidity, and length of hospital stay [2, 10, 11]. Today, in-hospital diabetes management is done by ward nurses who perform point-of-care (POC) glucose testing 3-5 times a day and rarely during the night. Diabetes is often a secondary diagnosis rather than the primary cause for admission, and patients are therefore under the care of non-diabetes specialists [12]. Improved in-hospital diabetes management strategies are greatly needed.

A combination of in-hospital diabetes teams, continuous glucose monitoring (CGM), and operational insulin titration algorithms to act on CGM data might contribute to this need. CGM measures glucose levels every 1-15 minutes, which might more accurately guide the titration of insulin therapy compared to (POC) glucose testing. CGM is commonly used in an *out-*hospital setting for patients with type 1 diabetes and has revolutionised diabetes management and increased patient satisfaction [13]. Recently, the COVID-19 pandemic has spurred interest in using *in*-hospital telemetric CGM to minimise the spread of infectious particles and reduce the usage of personal protection equipment [14]. The telemetric CGM system includes i) a CGM collecting real-time glucose data, ii) a smartphone in the patient room with a CGM application receiving the glucose data from the CGM via Bluetooth, and iii) a tablet in the nurse station receiving glucose data from the smartphone via an internet connection [15].

In-hospital CGM studies have mostly been observational and focused on the accuracy of CGMs versus POC glucose testing ﻿and, importantly, are mainly performed in an intensive care unit setting [16–20]. However, data on in-hospital CGM on glycaemic and clinical outcomes are scarce [21]. A recent review of five smaller RCTs on non-critically ill hospitalised patients with diabetes (*N*=291 in all five RCTs) indicates that in-hospital CGM is associated with a clinically insignificant reduction of mean daily glucose levels and increased detection of hypoglycemia of glucose levels <3 mmol/l [<54 mg/dl] compared with POC glucose testing [22]. Recent in-hospital RCTs find a small reduction of recurrent hypoglycaemic events [23] using CGM compared to POC glucose testing or no effect [24]. The outstanding results from the out-hospital setting on CGM use remain to be seen in an in-hospital setting. This might be because educated personnel, e.g., in-hospital diabetes teams with CGM competencies, and operational insulin titration algorithms might be imperative in achieving optimal use of telemetric CGM [12, 25–28]. In-hospital diabetes teams are cost-effective and are associated with a reduction in length of hospital stay, reduced in-hospital complications, and improved patient satisfaction [12, 29–31].

Until now, no RCTs have been investigating the effects of telemetric CGM with support from in-hospital diabetes teams with operational algorithms to act on CGM data.

## Methods

This DIATEC clinical investigation plan summary follows the SPIRIT guidelines [32].

### Trial design and population

**Fig. 1:**
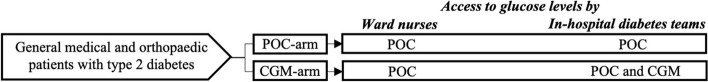
Design of the DIATEC trial

The DIATEC trial is a two-armed, two-site, prospective, randomised, open-label, blinded-endpoint (PROBE) trial (Fig. 1). In both arms (a POC-arm and a CGM-arm), hospitalised patients are monitored by POC glucose testing five times daily. Diabetes management is done by ward nurses guided by in-hospital diabetes teams. The in-hospital diabetes teams have operational algorithms for the titration of basal, prandial, and correctional insulin in both arms. In the POC-arm, insulin titration is guided by only POC glucose measurements. In the CGM-arm, insulin titration is guided by CGM data only, accessed by the in-hospital diabetes teams.

#### POC-arm

Ward nurses and the in-hospital diabetes teams can only access POC glucose testing for diabetes management. A blinded CGM is mounted for outcome analysis.

#### CGM-arm

Ward nurses have access to POC glucose testing only. The in-hospital diabetes teams have access to POC glucose testing and real-time CGM data for diabetes management.

**Fig. 2:**
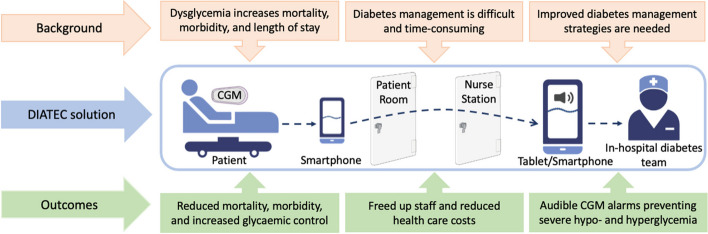
Telemetrics CGM setup and rationale of the DIATEC trial

Figure 2 Telemetrics CGM setup and rationale of the DIATEC trial. The orange section depicts the background of the DIATEC trial. The blue section depicts the telemetric CGM setup: From the left is a CGM-monitored patient, in the middle is the telemetric CGM setup, and to the right is the in-hospital diabetes teams managing patients with diabetes by CGM and operational insulin titration algorithms. The green section depicts the potential positive trial outcomes.

### Recruitment and time schedule

Recruiting takes place at the Copenhagen University Hospitals of North Zealand (NOH) and Herlev-Gentofte (HGH) in Denmark. First patient first visit was on 11^th^ April 2023. Last patient last visit is expected in October 2024.

### Inclusion, exclusion, and withdrawal criteria

#### Inclusion criteria

Type 2 diabetes prior to admission AND age ≥ 18 years old AND willingness and ability to comply with the clinical investigation plan AND ability to communicate with the trial personnel AND an expected length of hospital stay for at least two days after enrolment.

#### Exclusion criteria

Patients on out-hospital basal insulin with a duration of action > 24 hours (insulin glargine 300 U/ml or insulin degludec) OR hydroxyurea (CGM contraindication) OR nutritional therapy (continuous enteral or parenteral feeding) OR pancreatic disease leading to glucose metabolism disturbancies OR systemic glucocorticoid treatment with prednisone equivalent dose >5 mg/day OR expected to require admission to the intensive-care unit OR anasarca (severe and general edema, CGM contraindication) OR patients in dialysis OR estimated glomerular filtration rate (eGFR) <15 mL/min/1,73 m^2^ OR pregnant woman OR known hypersensitivity to the band-aid of the Dexcom G6 sensor.

#### Withdrawal criteria

Withdrawal of consent to participate OR transfer to intensive units OR transfer to other hospitals than NOH or HGH OR patients developing diabetic ketoacidosis OR patients developing hyperosmolar hyperglycaemic syndrome OR no longer fulfilling inclusion criteria OR any criterion equivalent to an exclusion criterion.

### In-hospital diabetes management

On admission, non-insulin antidiabetics are paused and patients are treated with basal insulin (0.20–0.25 U/kg) and correctional insulin (sliding scale insulin) in both the POC-arm and CGM-arm according to Danish national guidelines [33]. Prandial insulin (0.20–0.25 U/kg divided into three main meals according to international guidelines [31]) is added at the discretion of the in-hospital diabetes teams. Prandial insulin is withhold if patients are expected to eat less than 50% of the meal, if glucose levels are <3.9 mmol/l [<70 mg/dl], or during fasting. Insulin glargine 100 U/ml (Semglee) is used as basal insulin and insulin aspart Sanofi is used as prandial and correctional insulin.

We have developed operational algorithms (Table 1) for insulin titration aiming for standard in-hospital glucose levels of 5.6–10.0 mmol/l (100–180 mg/dl) in both arms [31, 33–36]. Generally, basal insulin doses are increased if nocturnal, i.e., fasting, hyperglycaemia is persistently observed. Prandial and correctional insulin doses are increased if daytime, i.e. non-fasting, hyperglycaemia is persistently observed, and vice versa regarding hypoglycaemia.

**Table 1:** Algorithm for titrating basal, prandial, and correctional insulin

**Orders at admission**			
Discontinue the patient’s oral and non-oral glucose-lowering medicaments.			
**Insulin orders during admission**			
**Basal insulin** *(mandatory order)*			
Basal insulin is given at lunch to allow same-day insulin titration decisions during the morning to take effect. Starting basal insulin dose is 0.25 U/kg, rounded to the nearest unit. Exceptions are:			
• Basal insulin dose is 0.20 U/kg in patients aged >75 years and/or with an eGFR ≤60 ml/min/1.73 m^2^ and/or body mass index (BMI) ≤ 22.5.			
• Basal insulin is continued on the usual out-hospital dose in patients treated with basal insulin at home.			
• In perioperative or fasting patients, basal insulin is paused on the day of surgery or during fasting and patients are managed by glucose-insulin-potassium (GIK) infusion. If the postoperative patient eats a full meal before 22:00 h, GIK infusion is stopped and half basal insulin dose is given. The full basal insulin dose is continued the following days.			
**Prandial insulin** *(ordered at the discretion of the in-hospital diabetes teams when glucose levels >10.0 mmol/l [>180 mg/dl] are observed postprandially for 1-2 days)*			
Starting total prandial insulin dose is 0.25 U/kg, rounded to the nearest unit, and given in three equally divided doses for breakfast, lunch, and dinner. Exceptions are:			
• Prandial insulin dose is 0.20 U/kg in patients aged >75 years and/or with an eGFR ≤60 mL/min/1.73 m^2^ and/or body mass index (BMI) ≤22.5.			
• Prandial insulin is continued on the usual out-hospital dose in patients treated with prandial insulin at home.			
• In perioperative or fasting patients, prandial insulin is paused on the day of surgery or during fasting.			
**Correctional insulin** *(mandatory order)*			
Glucose Level		Recommended insulin (U)	Insulin change (U)
mmol/l	mg/dl		
< 10.0	< 180	0	0
10.0 – 11.9	180 – 214	4	± 2
12.0 – 15.9	215 – 286	6	± 3
16.0 – 19.9	287 – 358	8	± 4
≥ 20.0	> 358	10	± 6
**Titrating basal, prandial, and correctional insulin**			
**Titrating basal and prandial insulin in the POC-arm**			
Titration of basal insulin (mmol/l) [mg/dl] is done daily by POC glucose levels during the night from 00:00 h till breakfast and is done prior to administration at lunch. Titration of prandial insulin for a specific meal is done by the pre-prandial POC glucose levels of the subsequent meal and for dinner, the POC glucose level before bedtime (22:00 h):			
• < 3.0 [< 54]: Decrease insulin dose by 30%.			
• 3.0 – 3.8 [54 – 69]: Decrease insulin dose by 20%.			
• 3.9 – 5.5 [70 – 99]: Decrease insulin dose by 10%.			
• 5.6 – 7.8 [100 – 140]: No changes.			
• 7.9 – 10.0 [141 – 180]: Increase insulin dose by 10% if no hypoglycaemic events are observed the previous day.			
• 10.1 – 15.0 [181 – 270]: Increase insulin dose by 20% if no hypoglycaemic events were observed the previous day.			
• >15.0 [>270]: Increase insulin dose by 30% if no hypoglycaemic events were observed the previous day.			
**Titrating prandial insulin in the CGM-arm**			
Prandial insulin is titrated by the nadir of the between-meal CGM glucose levels. If there is no nadir on the CGM trace between meals, the pre-prandial glucose level guides the decision to titrate prandial insulin. Titration of prandial insulin is guided by the glucose level ranges similar to titration of prandial insulin in the POC-arm.			
**Titrating basal insulin in the CGM-arm**			
Titration of basal insulin (mmol/l) [mg/dl] is done by glucose level ranges from 00:00 h till breakfast:			
• <3.0 [< 54]: If ≥5% of glucose levels are in this range and/or at least one level 2 hypoglycaemic event (three consecutive CGM glucose levels < 3.0 mmol/l [<54 mg/dl] [37]), decrease insulin dose by 30%.			
• 3.0 – 3.8 [54 – 69]: If ≥5% of glucose levels are in this range and/or at least one level 1 hypoglycaemic event (three consecutive glucose levels of 3.0–3.8 mmol/l [54–69 mg/dl] on CGM [37]), decrease insulin dose by 20%.			
• 3.9 – 5.5 [70 – 99]: If ≥10% of glucose levels are in this range, decrease insulin dose by 10%.			
• 5.6 – 7.8 [100 – 140]: No changes.			
• 7.9 – 10.0 [141 – 180): If ≥10% of glucose levels are in this range, increase insulin dose by 10%.			
• 10.1 – 15.0 [181 – 270]: If ≥10% of glucose levels are in this range, increase insulin dose by 20%.			
• >15.0 [>270]: If ≥10% of glucose levels are in this range, increase insulin dose by 30%.			
**Titrating correctional insulin in the POC-arm and CGM-arm**			
Titration of sliding scale insulin is done based on the decision of whether the patient is insulin resistant (add insulin) or insulin sensitive (subtract insulin) by adjusting levels of Recommended insulin (U) by the column Insulin change (U). The trend in increasing or decreasing basal and/or prandial insulin might guide this decision.			

For CGM-arm patients, real-time hypo- and hyperglycaemic audible CGM alarms are turned on in the in-hospital diabetes teams’ nurse stations on tablets. When the in-hospital diabetes teams are on ward rounds, they are equipped with a smartphone with the same alarm setup as the tablets. Hypoglycaemic alarms occur when glucose levels are <3.9 mmol/l [<70 mg/dl]. POC glucose testing must confirm hypoglycaemia on CGM due to reduced CGM sensor accuracy during hypoglycaemia [19]. Hyperglycaemic alarms occur when glucose levels are >13.9 mmol/l [>250 mg/dl] for two hours to avoid alarm fatigue. To avoid insulin stacking, hyperglycaemia on CGM is only corrected by warning of hyperglycaemic alarms and not by random viewing of CGM glucose levels. For POC-arm patients, hypo- and hyperglycaemia detected by POC glucose testing are corrected when observed.

POC-arm and CGM-arm patients are peroperatively monitored by POC glucose testing only. During fasting perioperatively (or insufficient food intake), patients are managed by glucose-insulin-potassium infusion [38]. When the patient eats a full meal again, insulin regimens are resumed.

From approx. 15:00 h to 08:00 h, and at weekends, the in-hospital diabetes teams are unavailable, and diabetes management is performed by ward nurses by POC glucose testing only in both arms.

### Titrating basal, prandial, and correctional insulin

Basal, prandial, and correctional insulin are titrated daily by the in-hospital diabetes teams in the POC-arm and CGM-arm. Titration is done by operational algorithms (Table 1) from 24-hour retrospective CGM data or POC glucose levels.

The three rules below apply for titrating basal and prandial insulin in both arms, aiming for glucose levels of 5.6–7.8 mmol/l [100–140 mg/dl]:

- *Rule of the lowest*: If glucose levels both *below and above* the range of 5.6–7.8 mmol/l [100 – 140 mg/dl] in Table 1 are observed during a titration period (and for CGM-arm patients above the listed percentage thresholds for decreasing or increasing basal insulin in Table 1, respectively), the lower glucose levels and hereby lowering of basal and/or prandial insulin must be prioritised.
- *Rule of the extremes*: The extremes of glucose levels *all below* or *all above* or *within and above* or *within and below* the range of 5.6–7.8 mmol/l [100–140 mg/dl] are prioritised in Table 1. For example, for CGM-arm patients, if ≥5% of glucose levels are <3.0 mmol/l [<54 mg/dl] and ≥5% of glucose levels are in the range of 3.0–3.8 mmol/l [54–69 mg/dl] during a titration period, the Table 1 recommendations for ≥5% of glucose levels <3.0 mmol/l [<54 mg/dl] (i.e., the “extreme” furthest away from the range of 5.6–7.8 mmol/l [100–140 mg/dl]) are followed.
- *Rule of missingness*: If all CGM data are temporarily missing (e.g., due to missing signal between the smartphone and CGM) or of unusable quality (e.g., due to multiple pressure-induced sensor attenuations), titration of insulin in CGM-arm patients is guided by POC glucose levels.

### Primary outcome

We assess the difference between arms regarding time in range (TIR), i.e., percentage of time during admission with glucose levels of 3.9–10.0 mmol/l [70–180 mg/dl] [37].

### Secondary outcomes

#### Glycaemic outcomes

Outcomes will be reported according to the newest CGM consensus [39]. Outcomes include (mmol/l) [mg/dl]: TIR per day 3.9–10.0 [70–180], time above ranges (TAR) >10.0 [>180], TAR >13.9 [>250], time below range (TBR) <3.9 [<70], TBR <3.0 [<54], and mean glucose levels. Glycaemic variability is reported as the CGM glucose distribution's standard deviation (SD) and coefficient of variation (CV). We report events, i.e. three consecutive CGM glucose levels, of hypoglycaemia <3.9 [<70], hypoglycaemia (level 1) 3.0–3.8 [54–69], hypoglycaemia (level 2) < 3.0 [<54], and the number of reoccurring hypoglycaemic events after the first episode of hypoglycaemia.

#### Clinical outcomes

We assess the length of hospital stay, any in-hospital related complications occurring at least one day after randomisation (e.g. acute kidney injury, death during hospitalisation, transfer to intensive care unit, etc.), the number of times basal, prandial, and correctional insulin is given in total *and* correctly given (according to Table 1 specifications), daily insulin doses, and readmission and death 30 days after hospitalisation.

### Tertiary outcomes

We assess the level of satisfaction with in-hospital CGM among the in-hospital diabetes teams and patients by questionnaires. Exploratory, we will identify which patients benefit from the in-hospital CGM setup (Fig. 2) in regard to glycaemic outcomes. We also evaluate how often the titration algorithm (Table 1) is followed correctly or not for both basal, prandial, and correctional insulin in both arms. The effects of the in-hospital diabetes teams are evaluated by comparing outcomes between periods when the in-hospital diabetes team are at work and off shifts.

### Data collection and trial procedures

A flowchart of trial procedures is provided in the Supplementary Table.

### Glucose data

Glucose data are collected by the CE-marked CGM Dexcom G6 (Dexcom Inc., San Diego, USA). The accuracy of in-hospital CGM use has been verified in previous studies [20].

### Demographic- and clinical data

The following data are recorded: Date of birth, ethnic origin, gender, height, and body weight at the time of admission. Blood pressure, pulse, oxygen saturation, and respiratory rate will be used to assess the National Early Warning Score [40] at inclusion.

### Medical history, medications, and concomitant disease

Information from patients’ electronic health records regarding co-morbidities, medical history, and intercurrent disease is registered at baseline and throughout the course of the trial.

### Laboratory tests

Blood samples upon inclusion include haemoglobin A1c, admission glucose, estimated glomerular filtration rate, and daily C-reactive protein measurements.

### Questionaries

Questionaries about the patient’s experiences with having their diabetes managed by in-hospital diabetes teams and if the CGM has been troublesome in any way are developed from The Diabetes Treatment Satisfaction Questionnaire for Inpatients [41]. Questionnaires for the in-hospital diabetes team members on whether CGM is an advantage or not compared to POC glucose testing in managing in-hospital diabetes patients have been developed from previously used questionaries [15].

### Statistical considerations

Glycaemic outcomes are compared between arms for daytime and nighttime and the total day. Continuous variables are compared between arms by linear regression analysis. A zero-adjusted Gamma distribution accommodates the positivity and exact zero in the outcomes. Categorical variables are compared with the chi-squared test. Discrete variables are compared with Poisson regression or negative binomial regression. A linear mixed model is applied for repeated measures of daily insulin dose and C-reactive protein levels. In addition, a per-protocol analysis will be performed for glycaemic outcomes, including only CGM data from when the in-hospital diabetes teams are on shifts (and compared to when the in-hospital teams are not on shifts) and where expert decisions and appropriate actions on titrating insulin, according to Table 1 and correcting hypo- and hyperglycaemia are made. Missing data is handled by multiple imputation methods. Statistical significance is set at a two-sided *P*-value ≤0.05.

### The sample size for the primary outcome

From our own studies on patients with diabetes admitted with pneumonia at NOH, we know that the mean TIR (±SD) is approximately 60–70%±20–25%  [10, 15]. A recent in-hospital CGM trial found a TIR (±SD) of 50%±25% [23]. If the expected difference between arms on TIR is set at 10 percentage points, SD to 23%, statistical power to 80%, and an alfa level of 5%, the inclusion of 166 patients is required. With a dropout rate of 20%, we aim to include 208 patients.

### Early termination of the trial

The sponsor reserves the right to terminate the trial due to safety concerns and/or proven lack of efficacy. The sponsor and investigators should promptly inform the Medical Research Ethics Committees and patients and ensure appropriate follow-up in case of early termination of the trial.

### Data management plan

REDCap [42] is used as an electronic case report form (eCRF). Data is handled according to the General Data Protection Regulation and the Danish Data Protection Act. Data are stored for 10 years after the end of the trial according to the ISO 14155 standard. All investigators have full access to all non-blinded data during the trial and all data at the end of the trial in REDCap.

### Adverse events recording and reporting

Adverse events and device deficiencies are recorded on standardised forms in the eCRF and followed up as appropriate.

### Randomisation

Randomisation is performed in the eCRF by a blocked randomisation list to ensure equal distribution of patients between the sites NOH and HGH and between arms. The randomisation list was set up by independent personnel not involved in other parts of the trial.

## Discussion

Results will be presented at international conferences and in peer-reviewed scientific journals. The DIATEC trial will show the effects of in-hospital CGM in combination with diabetes teams and operational insulin titration algorithms to act on CGM data in managing hospitalised patients with type 2 diabetes. This might constitute a new in-hospital diabetes management strategy with improved patient prognosis and healthcare resource savings worldwide. The DIATEC trial seeks to identify which hospitalised patients will benefit from CGM and an in-hospital diabetes team which is invaluable information to optimise the use of healthcare resources before broadly implementing in-hospital CGM and diabetes teams.

### Supplementary Information

**Supplementary Material 1.**

**Supplementary Material 2.**

## Data Availability

The datasets generated and/or analysed during the DIATEC trial are available from the corresponding author upon reasonable request or published with future publications.

## Abbreviations

CE: Conformité européenne
CGM: Continuous glucose monitoring
CV: Coefficient of variation
eCRF: electronic case-report form
HbA1c: Haemoglobin A1c
HGH: Copenhagen University Hospital – Herlev-Gentofte
NOH: Copenhagen University Hospital – North Zealand
POC: Point-of-care
PROBE: Prospective randomised open-label blinded endpoint
RCT: Randomised controlled trial
SD: Standard deviation
SPIRIT: Standard protocol items: recommendations for interventional trials
TAR: Time above range
TBR: Time below range
TIR: Time in range
