# Supplementary material for: Summary of clinical investigation plan for The DIATEC trial: in-hospital diabetes management by a diabetes team and continuous glucose monitoring or point of care glucose testing – a randomised controlled trial
Source: BMC Endocr Disord. 2024 May 6;24:60. doi: 10.1186/s12902-024-01595-4 (PMC11071255; doi:10.1186/s12902-024-01595-4)
Supplement: Supplementary file 2 — Supplementary Material 2. [file 12902_2024_1595_MOESM2_ESM.docx]

**Supplementary Table.** Schedule of trial procedures.

| **Visit** | **Screening** | | **First visit** | **Daily visits** | **Intermediate visits** | **Last visit** |
| --- | --- | --- | --- | --- | --- | --- |
| **Days** | **Day 0** | | **Day 1** | **Between days 1 and 10** | **Ever 10^th^ day** | **End of trial** |
| **PROCEDURES** | | | | | | |
| Compliance with in- and exclusion criteria | | X | X |  |  |  |
| Informed written consent | |  | X |  |  |  |
| Randomisation | |  | X |  |  |  |
| Medical history and concomitant diseases | |  | X | X | X | X |
| Concomitant and previous medication | |  | X | X | X | X |
| Vital signs | |  | X |  |  |  |
| Demography | |  | X |  |  |  |
| Blood samples | |  | X | X | X | X |
| Dexcom G6 CGM mounting/replacement | |  | X |  | X |  |
| Install telemetric CGM setup | |  | X |  |  |  |
| Collect smartphone, CGM, and questionaries | |  |  |  |  | X |
| **IN-HOSPITAL DIABETES MANAGEMENT** | | | | | | |
| Registration of glucose levels^*^ | |  | X | X | X | X |
| Registration of insulin doses | |  | X | X | X | X |
| Insulin titration | |  | X | X | X | X |
| **SAFETY ASSESMENT**^†^ | | | | | | |
| Assessment of safety parameters | |  | X | X | X | X |
| Telemetric CGM accountability | |  | X | X | X | X |

* Registration of CGM glucose levels is done daily in the electronic health record, indicating the percentage of time in the different blood sugar ranges listed in Table 1.
^†^ Safety procedures are performed daily by the in-hospital diabetes teams and include that the correct insulin dose is administered and the telemetric CGM setup is running.
